# Working Characteristics of Variable Intake Valve in Compressed Air Engine

**DOI:** 10.1155/2014/498934

**Published:** 2014-10-14

**Authors:** Qihui Yu, Yan Shi, Maolin Cai

**Affiliations:** School of Automation Science and Electrical Engineering, Beihang University, Beijing 100191, China

## Abstract

A new camless compressed air engine is proposed, which can make the compressed air energy reasonably distributed. Through analysis of the camless compressed air engine, a mathematical model of the working processes was set up. Using the software MATLAB/Simulink for simulation, the pressure, temperature, and air mass of the cylinder were obtained. In order to verify the accuracy of the mathematical model, the experiments were conducted. Moreover, performance analysis was introduced to design compressed air engine. Results show that, firstly, the simulation results have good consistency with the experimental results. Secondly, under different intake pressures, the highest output power is obtained when the crank speed reaches 500 rpm, which also provides the maximum output torque. Finally, higher energy utilization efficiency can be obtained at the lower speed, intake pressure, and valve duration angle. This research can refer to the design of the camless valve of compressed air engine.

## 1. Introduction

Environmental issues such as fog, haze, greenhouse effect, and acid rains have been widely concerning. Burning of fossil fuels in internal combustion engines (ICE) for transportation is the major source of environmental issues [1–3]. New energy sources such as wind, solar energy, compressed air which can replace the fossil fuel are an obvious solution to solve environment issues [4]. With respect to environmental protection, the issue of energy expenditure has been emphasized [5]. Some scholars believe traditional automobiles will be replaced by new energy vehicles in the future. So far, there are some new energy vehicles, namely, electric vehicles, hybrid electric vehicles, compressed air engines (CAE), and so on. The CAE is the typical product of zero-pollution vehicles, which has been studied by many scholars and institutions [6].

To ensure smooth running and fast response, the flow of air is controlled by a simple cam mechanism in many CAE systems [7–9]. Conventional mechanical valve trains generally use valve timings and lifts which are fixed depending upon cam mechanism design. The lack of flexibility of camshaft based valve trains to vary timing, duration, and lift of intake valves is one of the disadvantages [10]. Because the CAE does mechanical work by expanding compressed air, the flow of compressed air must be controlled to improve energy efficiency. It is obvious that the cam mechanism is difficult to meet the demand. In order to optimize energy efficiency, the variable intake valve techniques have been used in the CAE [11].

The variable intake valve techniques have the potential to be widely used in internal combustion engines to reduce energy losses and fuel consumption [12–17]. Previous studies have mainly focused on simulations and system integrations based on cam mechanism valve. Few studies have been reported about the variable intake valve investigations in CAE.

This paper focuses on the influences on the performance of the CAE by the variable intake valve lift and duration. Thus, detailed mathematical models to describe the working process are built and verified by experiments. This paper is organized as follows. In Section 2, detailed mathematical models are discussed. In Section 3, simulation and real experiments results are obtained and compared to verify the accuracy of the theoretical models. In Section 4, the influences on the performance of the CAE by the lift and duration of the variable intake valve are analyzed. Finally, conclusions are presented in Section 5.

## 2. Theoretical Analysis

**Figure 1:**
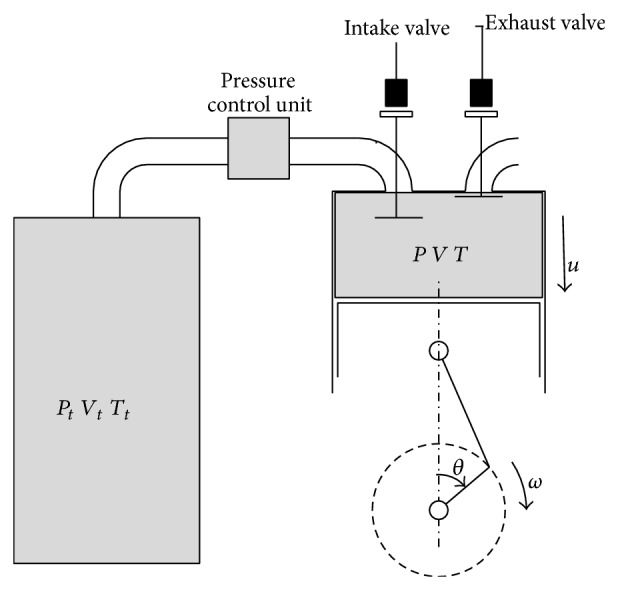
Cylinder-tank model.

To understand the working process of the CAE, we need to study the in-cylinder process, which is illustrated in Figure 1. The gas tank provides energy source. The intake pressure is regulated by pressure control unit. Air flow is controlled by solenoid valve. There are mainly three components: the cylinder, the valves, and the tank. In the following, we build these models based on thermodynamics and piston kinematics. For a single-stage piston-type CAE, compressed air enters the cylinder through the intake valve and the piston is pushed by compressed air. Then the intake valve closed after a specific crank angle, while the compressed air continues to push the piston down and output work. When the piston reaches the bottom dead center (BDC), the exhaust valve opens so that the air with residual pressure discharges. The piston moves from the BDC to the top dead center (TDC); the CAE completes a work cycle.

### 2.1. Valve Flow

Because the throttling effect from the intake or exhaust valve accounts for energy losses, valve flow is critical to the CAE. Valve flow is considered as one-dimensional isentropic flow [18, 19].

If *p*_*d*_/*p*_*u*_ > *b*, the mass flow rate is given by

(1) $$\begin{array}{c} G=Ap_{u}\sqrt{\frac{2k}{\left(k-1\right)R\theta_{u}}\left[{\left(\frac{p_{d}}{p_{u}}\right)}^{2/k}-{\left(\frac{p_{d}}{p_{u}}\right)}^{\left(k+1\right)/k}\right]}. \end{array}$$

If *p*_*d*_/*p*_*u*_ ≤ *b*, the flow is choked, and the mass flow rate is given by

(2) $$\begin{array}{c} G=Ap_{u}\sqrt{\frac{k}{R\theta_{u}}{\left(\frac{2}{k+1}\right)}^{\left(k+1\right)/\left(k-1\right)}}, \end{array}$$

where *b* = (2/*k*+1)^*k*/*k*−1^ is upstream stagnation sound speed.

The valve flow area is represented by *A*, which can be expressed by the following equation:

(3) $$\begin{array}{c} A=C_{d}A_{v}. \end{array}$$

The relationship between the valve flow area and the valve lift is defined by the following equation:

(4) $$\begin{array}{c} A_{v}=aL_{v}. \end{array}$$

The scale factor “*a*” is defined by

(5) $$\begin{array}{c} a=\frac{A_{vm}}{\text{IVL}}, \end{array}$$

where *A*_*vm*_ is the maximum valve flow area.

We can characterize the camless valve motion by angle (or opening) IVO, maximum lift IVL, and duration IVD of each intake valve. For simplicity, the camless intake and exhaust valve lift profile model is presented by the following equations:

(6) $$\begin{array}{c} L_{v}=\{\begin{array}{cc} s_{r}\left(t-t_{1}\right) & t\geq t_{1},\;\;t<t_{2}\\ \text{IVL} & t\geq t_{2},\;\;t<t_{3}\\ \text{IVL}-s_{c}\left(t-t_{3}\right) & t\geq t_{3},\;\;t<t_{4}\\ 0 & \text{otherwise}, \end{array} \end{array}$$

where

(7) $$\begin{array}{c} t_{1}=t_{\text{IVO}},\;\;t_{2}=t_{1}+t_{r},\\ t_{3}=t_{2}+t_{\text{IVD}},\;\;t_{4}=t_{3}+t_{c}. \end{array}$$

**Figure 2:**
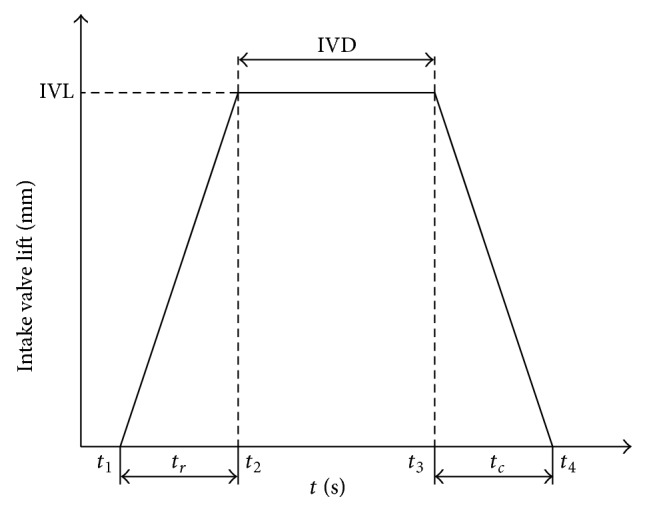
Valve lift profile.

*s*_*r*_ and *s*_*c*_ are fixed in the time domain. A coordinate transformation to crank angle domain results in different valve profiles at different engine speeds. The valve lift profile is shown in Figure 2.

### 2.2. In-Cylinder Process

The cylinder content is energy exchange process. The pressure and temperature of compressed air inside the cylinder are calculated by a global energy balance:

(8) $$\begin{array}{c} \frac{dU}{dt}=\frac{dq}{dt}+\frac{dm_{i}}{dt}h_{i}-\frac{dW}{dt}-\frac{dm_{e}}{dt}h_{e}, \end{array}$$

where *dU*/*dt* is the rate of the internal energy of the air inside the cylinder, *dq*/*dt* is the rate of heat transferred from the cylinder wall to the cylinder contents, and *dW*/*dt* is the rate of work done by the open system (which is equal to *pdV*/*dt*).

The internal energy of the air can be expressed as

(9) $$\begin{array}{c} \frac{dU}{dt}=\frac{d\left(mu\right)}{dt}=m\frac{du}{dt}+u\frac{dm}{dt}, \end{array}$$

where *u* = *C*_*v*_ *θ*, *m* = *m*_*i*_ − *m*_*e*_.

Substituting (9) into (8) yields

(10) $$\begin{array}{c} \frac{d\theta }{dt}=\frac{1}{mC_{v}}\left[\frac{dq}{dt}+h_{i}G_{i}-h_{e}G_{e}-p\frac{dV}{dt}-uG\right], \end{array}$$

where *G*_*i*_ = *dm*_*i*_/*dt*, *G*_*e*_ = *dm*_*e*_/*dt*, *G* = *dm*/*dt*.

The rate of the pressure change inside the cylinder is obtained by the ideal gas law:

(11) $$\begin{array}{c} p\frac{dV}{dt}+V\frac{dp}{dt}=mR\frac{d\theta }{dt}+R\theta \frac{dm}{dt}. \end{array}$$

### 2.3. Heat Transfer

In order to evaluate the instantaneous heat interaction between the cylinder content, the heat transfer coefficient *K*_*w*_ must be defined. According to literature [20], assuming that the gas velocity is proportional to the average piston speed *U*_*p*_, the heat transfer coefficient *K*_*w*_ can be expressed with the following equation:

(12) $$\begin{array}{c} K_{w}=\left(0.1129\right)d^{-0.2}p^{0.8}U_{p}^{0.8}\theta^{-0.594}. \end{array}$$

The average piston speed can be expressed by the following equation:

(13) $$\begin{array}{c} U_{p}=\frac{S\cdot n}{30}. \end{array}$$

The corresponding heat transfer is

(14) $$\begin{array}{c} \frac{dq}{dt}=K_{w}A_{w}\left(\theta_{w}-\theta \right), \end{array}$$

where the total surface area *A*_*w*_ can be expressed with crank angle as follows:

(15) Aw(φ)=π2D2+π2DS ×[1−cos⁡φ+1λ(1−1−λ2sin2(φ))].

### 2.4. Piston Ring Friction

The differential element of friction work *δW*_*f*_ for the compression ring can be expressed as

(16) $$\begin{array}{c} \delta W_{f}=\mu_{r}p\pi Dd_{r}\delta S, \end{array}$$

where *δS* is piston stroke through which this force acts.

This expression is integrated over a complete engine cycle to account for the work lost to friction, which is then subtracted from the net cycle work.

## 3. Simulation and Experimental Validation

### 3.1. Simulation of the CAE

The working characteristics of the CAE are determined by the theoretical analysis mentioned in Section 2. The nonlinear and coupled differential equations are modelling in MATLAB/Simulink.  Table 1 shows the initial values of the parameters.

**Figure 3:**
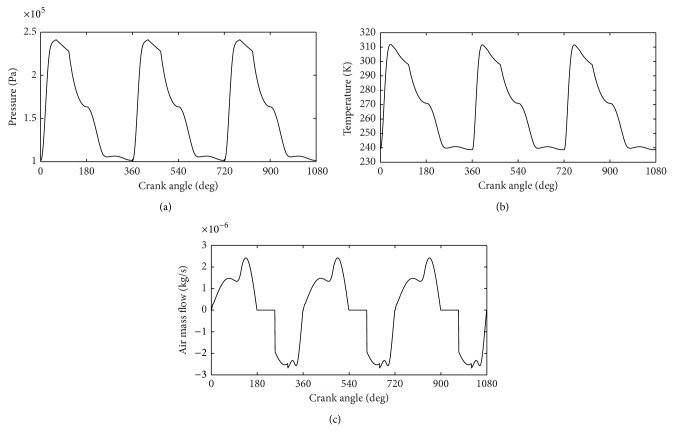
Pressure curve, temperature curve, and mass curve of the cylinder.

Figures 3(a), 3(b), and 3(c) show the simulation results. The air pressure of the cylinder is shown in Figure 3(a), the air temperature of the cylinder is plotted against the crank angle in Figure 3(b), and Figure 3(c) depicts the air mass flow of the cylinder curve.

As shown in Figure 3, the pressure, temperature, and the mass inside the cylinder of the CAE change periodically. The intake valve opens when the piston reaches the TDC; compressed air from high pressure tank rapidly flows into the cylinder. The pressure inside the cylinder rapidly increases to the intake pressure. Meanwhile, the mass and temperature inside the cylinder increase. When the mass flow rate is less than the rate of cylinder volume, the pressure of the cylinder drops dramatically. Meanwhile, the compressed air inside the cylinder expands and leads to the temperature of the cylinder drop from its peak.

Compressed air no longer flows into the cylinder, when the intake valve is closed. At this time, the mass flow of air drops to zero. The piston is pushed to the BDC depending on compressed air inside the cylinder expansion. The temperature and pressure inside the cylinder drop dramatically.

The exhaust valve opens when the piston reaches the BDC. The residual compressed inside the cylinder is discharged, and the mass inside the cylinder decreases from its top. Meanwhile, the temperature and pressure inside the cylinder drop to their bottoms.

The above process is repeated and mechanical power can be output continuously.

According to Figure 3(b), the temperature of the cylinder reaches 240 K which may experience icing, so heat exchange must be used.

### 3.2. Experimental Verification

**Figure 4:**
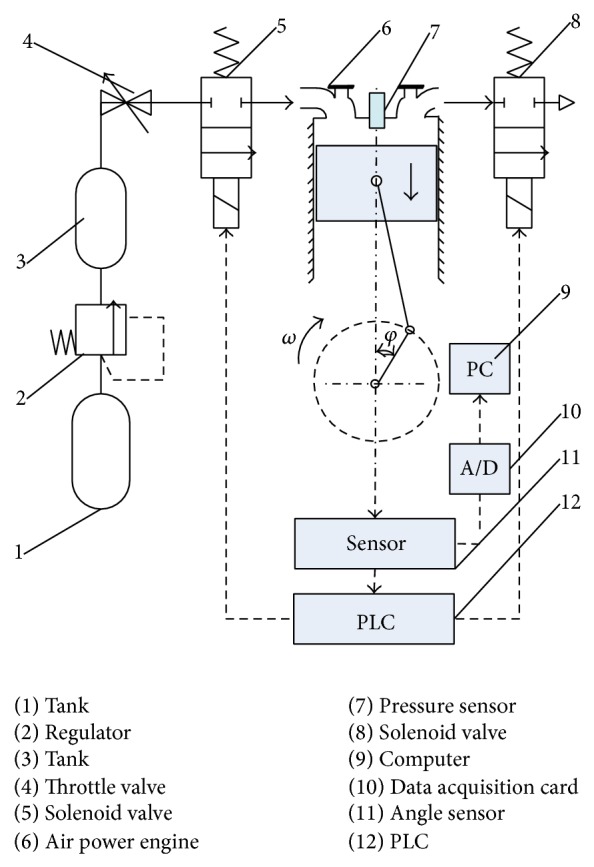
Configuration of experimental apparatus.

The experiments were conducted to verify the accuracy of the mathematical model. The experimental apparatus is shown in Figure 4, which consists of a high pressure tank, a regulator (IR3020-03BC), a low pressure tank, a throttle valve (AS3001F), two port solenoid valves, a refit engine with basic parameters shown in Table 2, a data acquisition card (PCI1711) by Advantech, an absolute angular displacement sensor, and program logic controller (PLC) by Siemens. In the experiment, a 4-stroke gasoline engine was reformed to a compressed air engine by the intake port and exhaust port solenoid valve. The engine specifications are shown in Table 2.

In this experiment, firstly, the compressed air source worked and the outlet pressure of the regulator was set to the fixed value. Secondly, the low pressure tank maintained the pressure after a period of time, then adjust throttle valve which can let compressed air exhausted steadily from the tank. The intake port and the exhaust port solenoid valves were controlled by PLC with shaft angle which was detected by absolute value of the angular sensor. The intake port solenoid valve opened when the piston reached the TDC and closed completely at a crank angle. Then, compressed air inside the cylinder expands. During this process, the exhaust port solenoid stayed closed, and the piston was pushed from the TDC toward the BDC by the incoming compressed air, producing the power stroke. The exhaust solenoid valve opened when the piston reaches the BDC. During the process, the intake solenoid valve remained closed. The compressed air inside the cylinder was discharged from the cylinder, and the piston moved from the BDC towards the TDC. The crank angle was measured by absolute value of the angular displacement sensor. The last stage was data acquisition and storage.

**Figure 5:**
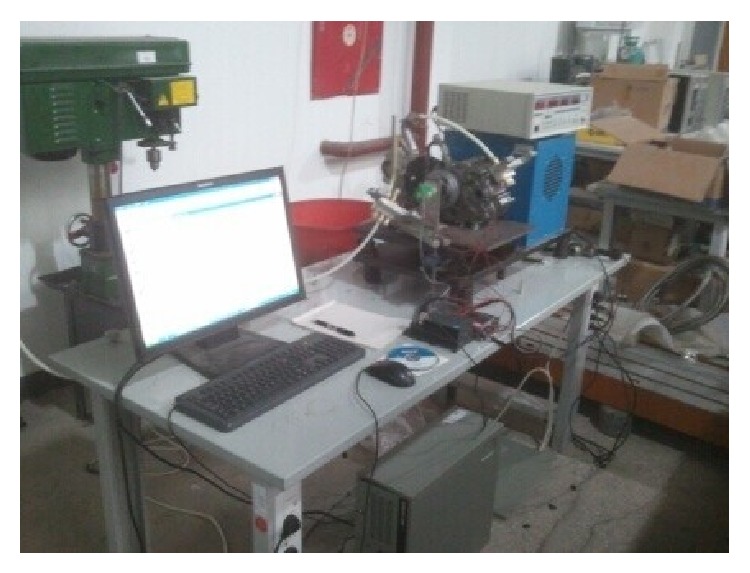
The experiment of air powered engine.

The testing rig is built as shown in Figure 5. The main parameters of the cylinder are presented in Table 2.

**Figure 6:**
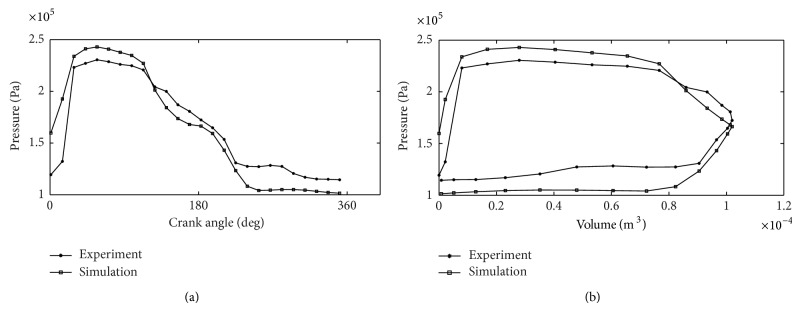
Experimental and simulation curves of cylinder pressure.

As shown in Figure 6, the simulation curve trend is consistent with the experimental curve trend, and the mathematical model above can be verified. However, there are three differences between the simulation results and the experimental results: (1) the maximum pressure is different; (2) the experimental curve is backward offset to simulation curve; (3) the experiment exhaust pressure value is greater than the simulation exhaust pressure value.

The main reasons for the differences are summarized as follows. Considering the small effective flowing area in the intake solenoid, the throttling effect will be quite evident. Meanwhile, each solenoid valve experiences delay in motion, but the delay time is different under different situation. In this paper, the simulation is based on the assumption that the delay time is constant for simplicity. Therefore, the experiment pressure curve is backward offset to simulation curve. And when the exhaust air mass flow is less than the rate of cylinder volume, the pressure inside the cylinder will increase during exhaust process.

**Figure 7:**
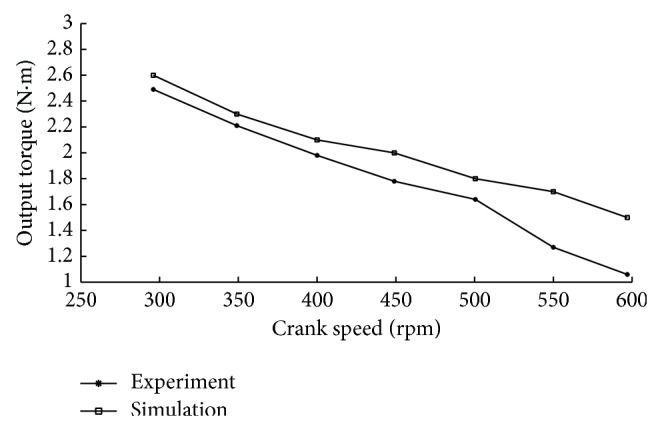
Experiment and simulation curves of output torque.

Experiment and simulation curves of output torque are shown in Figure 7. It is obvious that the experimental and simulation curves have similar trends. Both output torque curves decrease when the rotate speed increased. But throttling loss is not considered in the simulation process, so the output torque in the simulation is greater than the experiment value at different crank speeds. It is obvious that the differences between experimental and numerical results are increased with the crank speed increasing. That is because the bearings friction torque, auxiliaries, and gears torque losses are not considered in the numerical calculated. These torques will increase along with the increase of the crank speed.

## 4. Performance Analysis

Energy efficiency evaluation criterion to ICE is not suitable but not for the CAE. In this section, a new energy efficiency evaluation, namely, the air power, is briefly introduced to evaluate the energy efficiency of the CAE.

The air power is expressed using the available energy [21], which is expressed as

(17) $$\begin{array}{c} P=p_{a}Q_{a}\left[\ln\frac{p_{s}}{p_{a}}+\frac{k}{k-1}\left(\frac{\theta_{s}-\theta_{a}}{\theta_{a}}-\ln\frac{\theta_{s}}{\theta_{a}}\right)\right], \end{array}$$

where *Q*_*a*_ is the volume of air at the standard state.

The energy efficiency can be expressed by

(18) $$\begin{array}{c} \eta =\frac{P}{P_{m}}, \end{array}$$

where

(19) $$\begin{array}{c} P_{m}=\frac{\text{ IT }\cdot n}{9550}, \end{array}$$

where IT indicates torque.

The indicated torque can be expressed by

(20) $$\begin{array}{c} \text{IT}=\frac{\int pdV}{2\pi }. \end{array}$$

From the previous discussion, the performance of CAE can be obtained in different intake pressure, IVD, and IVL. The initial values of the parameters are shown in Table 1. Intake pressure, IVD, and IVL can be changed for comparison while all the other parameters are kept constant.

**Figure 8:**
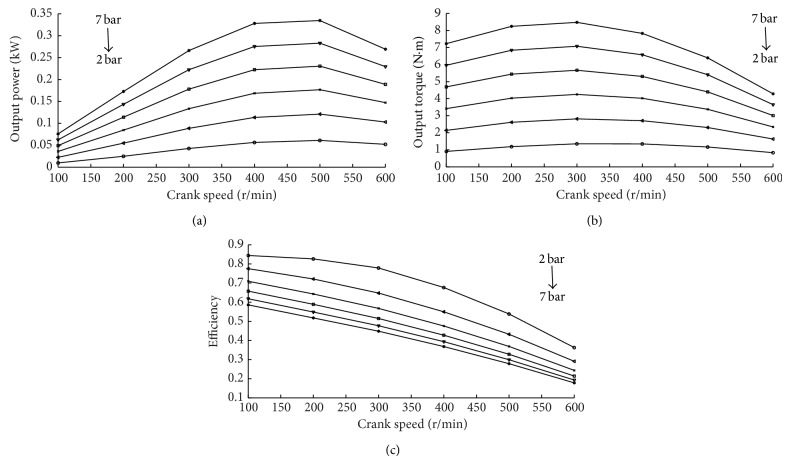
The relationship of intake pressure and performance of CAE.

Figures 8(a) and 8(b) show the power and torque output from the CAE at various supplied pressures. The highest power output of 0.3345 kW is obtained at 7 bar and 500 rpm. The highest torque output of 8.4727 Nm is obtained at 7 bar and 300 rpm. The highest supplied pressure will obtain the highest torque and power output.

The energy efficiencies under various intake pressures and crank speeds are shown in Figure 8(c). The lowest crank speed leads to the highest energy efficiency. And the lowest air pressure provides the highest efficiency.

It is clear that increasing supply pressure is beneficial to output more power and torque. However, the method will reduce energy efficiency.

**Figure 9:**
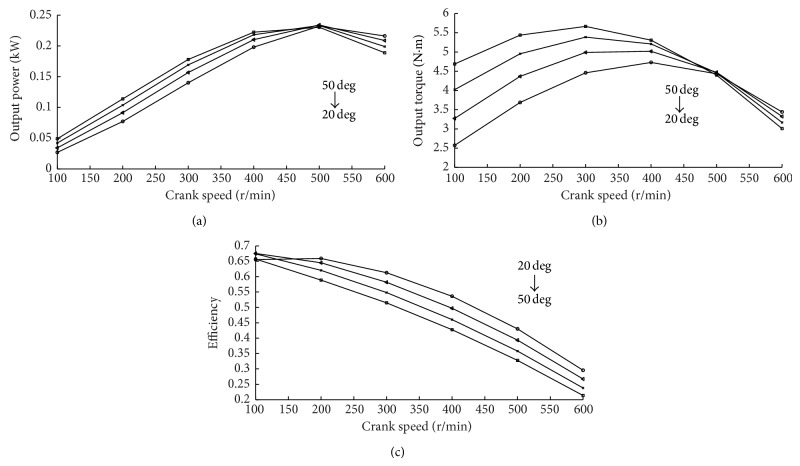
The relationship of IVD and performance of CAE.

Figure 9 shows the performance of the CAE in various IVD angles at 5 bar intake pressure.

The power and torque output from the CAE are obtained by simulation at various IVD angles, as shown in Figures 9(a) and 9(b). The highest power output is obtained at 500 rpm in any IVD angle. The output torque increases with the IVD. The output power and torque are equal in different IVD angle at 500 rpm. The energy efficiency would decrease with the IVD and can be expressed in Figure 9(c). But when IVD is equal to 20 deg, the efficiency will drop at crank speed of 100 rpm. That is because the more the compressed air enters into CAE at the lowest crank speed, the higher the pressure exhausts are.

**Figure 10:**
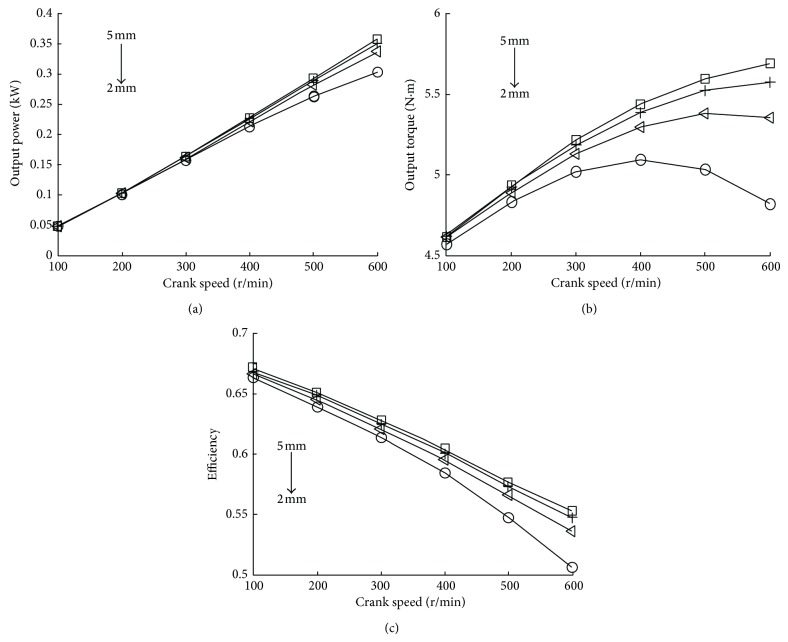
The relationship of IVL and performance of CAE.

Figure 10 shows the performance of the CAE in various IVL at 5 bar intake pressure.

The power and torque output from the CAE are obtained by simulation at various IVL, as shown in Figures 10(a) and 10(b). The output power increases with the crank speed. But when crank speed is lower than 400 rpm, the output power has little change at various IVD. That is because in low crank speed the air flow mass is almost stable with different IVL. Meanwhile, at the beginning, the output torque increases with the increase of the crank speed and reaches its peaks at different crank speeds and IVL. The energy efficiency would decrease with the crank speed and large IVL is beneficial to improve the energy efficiency which can be expressed in Figure 10(c). Throttling effect will decrease in large IVL.

## 5. Conclusions

In this paper, the mathematical model was built. Simulation and experimental studies on the CAE were done, and the conclusions are summarized as follows.

1. Compressed air pressure inside the cylinder and output torque have the same changing tendency in both simulation curve and experimental curve.
2. The highest power output is obtained at 500 rpm, and the highest torque output is obtained at 300 rpm at different intake pressures and different IVD angles.
3. When crank speed is higher than 200 rpm, higher energy utilization efficiency can be obtained at the lower speed, intake pressure, and IVD.
4. The output torque increases with the increase of the crank speed and reaches its peaks at different crank speeds and IVL. And large IVL is beneficial to improve the energy efficiency.

## Figures and Tables

**Table 1:** Initial value of the parameters.

Parameter	value
*D* (m)	0.05
*S* (m)	0.052
IVO (deg)	0
IVD (deg)	130
EVO (deg)	180
EVC (deg)	360
*p*_*s*_ (bar)	2.5
*b*	0.41
*C*_*m*_	2.3
*λ*	0.263
*n* (rpm)	500
*t*_*r*_ (s)	0.02
*C*_*d*_	0.8

**Table 2:** Engine specifications.

Engine model	DJ139FMA
Engine type	Single cylinder, 4-stroke, spark-ignited, air-cooled engine
Cylinder stroke/bore	50/52 mm
Displacement volume	100 cm^3^

## Nomenclature

A: Area (${\text{m}}^{2}$)
b: Critical pressure ratio
$C_{v}\text{:}$: Specific heat at constant volume (J/(kg·K))
$C_{p}\text{:}$: Specific heat at constant pressure (J/(kg·K))
$C_{d}\text{:}$: The valve discharge coefficient
D: Piston diameter (m)
E: Thermodynamic internal energy (J)
G: Air mass flow (kg/s)
h: The enthalpies of air (W/(${\text{m}}^{2}$K))
IVO: Valve lift opening angle (°CA)
IVL: Valve maximum lift (m)
IVD: Valve duration angle (°CA)
$K_{w}\text{:}$: Heat transfer coefficient
k: Specific heat ratio
L: Valve lift (m)
m: Air mass (kg)
n: Engine speed (rpm)
p: Pressure (Pa)
q: Heat exchange (J)
Q: The volume of air (${\text{m}}^{3}$/s)
R: Gas constant (J/(kg·K))
S: Piston stroke (m)
$s_{r}\text{:}$: Intake valve opening inclination
$s_{c}\text{:}$: Intake valve closing inclination
t: Time (s)
$t_{r}\text{:}$: The time for intake valve to reach maximum lift (s)
$t_{s}\text{:}$: The time for intake valve from maximum lift to closing (s)
U: The internal energy of the air (J)
u: Velocity (m/s)
V: Volume (${\text{m}}^{3}$)
W: Energy (J)
φ: Crank angle (rad)
$\mu_{r}\text{:}$: The sliding friction coefficient
η: Efficiency
ω: Crank speed (rad/s)
θ: Temperature (K).

### Subscripts

a: Atmosphere
d: Downstream side
i: Entering
e: Leaving
IVO: Redundant use of opening
IVD: Duration of intake valve opening
o: Valve
s: Supply of CAE
t: Tank
u: Upstream side
v: Valve.

## References

[1] Chen H., Ding Y., Li Y., Zhang X., Tan C. (2011). Air fuelled zero emission road transportation: a comparative study. *Applied Energy*.

[2] Singh B. R., Singh O. (2012). A study of performance output of a multivane air engine applying optimal injection and vane angles. *International Journal of Rotating Machinery*.

[3] Veziroglu T. N., Sahin S. (2008). 21st Century's energy: hydrogen energy system. *Energy Conversion and Management*.

[4] Morita K. (2003). Automotive power source in 21st century. *JSAE Review*.

[5] Chau K. T., Wong Y. S. (2002). Overview of power management in hybrid electric vehicles. *Energy Conversion and Management*.

[6] Ordonez C. A. (2000). Liquid nitrogen fueled, closed Brayton cycle cryogenic heat engine. *Energy Conversion and Management*.

[7] Huang C.-Y., Hu C.-K., Yu C.-J., Sung C.-K. (2013). Experimental investigation on the performance of a compressed-air driven piston engine. *Energies*.

[8] Da A. N., Jian T., Zuo C.-J. (2005). Designing and experiment ofa compressed-air engine. *Journal of Heifei University of Technology*.

[9] Trajkovic S., Tunestal P., Johansson B. (2008). Investigation of different valve geometries and valve timing strategies and their effect on regenerative efficiency for a pneumatic hybrid with variable valve actuation. *SAE International*.

[10] Saied S. A. G. M., Jazayeri S. A., Shamekhi A. H. Modeling of variable intake valve timing in SI engine.

[11] Christopher M. H., Lee D. (2014). Block: compressed air engine. *U.S. Patent*.

[12] Kumar R., Dixit A. K. (2014). Combustion and emission characteristics of variable compression ignition engine fueled with *Jatropha curcas* ethyl ester blends at different compression ratio. *Journal of Renewable Energy*.

[13] Izadi Najafabadi M., Abdul Aziz N. (2013). Homogeneous charge compression ignition combustion: challenges and proposed solutions. *Journal of Combustion*.

[14] Sher E., Bar-Kohany T. (2002). Optimization of variable valve timing for maximizing performance of an unthrottled SI engine-a theoretical study. *Energy*.

[15] Gölcü M., Sekmen Y., Erduranli P., Salman M. S. (2005). Artificial neural-network based modeling of variable valve-timing in a spark-ignition engine. *Applied Energy*.

[16] Fontana G., Galloni E. (2009). Variable valve timing for fuel economy improvement in a small spark-ignition engine. *Applied Energy*.

[17] Mahrous A.-F. M., Potrzebowski A., Wyszynski M. L., Xu H. M., Tsolakis A., Luszcz P. (2009). A modelling study into the effects of variable valve timing on the gas exchange process and performance of a 4-valve DI homogeneous charge compression ignition (HCCI) engine. *Energy Conversion and Management*.

[18] Shi Y., Cai M. (2011). Working characteristics of two kinds of air-driven boosters. *Energy Conversion and Management*.

[19] Wang X., Tsao T.-C., Tai C., Kang H., Blumberg P. N. Modeling of compressed air hybrid operation for a heavy duty diesel engine.

[20] Bossel U. (2005). Thermodynamic analysis of compressed air vehicle propulsion. *Journal of KONES, Internal Combustion Engines*.

[21] Cai M., Kawashima K., Kagawa T. (2006). Power assessment of flowing compressed air. *Journal of Fluids Engineering, Transactions of the ASME*.

